# Knowledge, Attitude and Practices (KAP) towards Diet and Health among International Students in Dublin: A Cross-Sectional Study

**DOI:** 10.3390/ijerph17093182

**Published:** 2020-05-03

**Authors:** Xiyao Liu, Haoyue Chen, Qianling Zhou, Huifeng Zhang, Phensiri Asawasirisap, John Kearney

**Affiliations:** 1 Department of Maternal and Child Health, School of Public Health, Peking University, Beijing 100191, China; liuxiyao1997@163.com (X.L.); haoyuechen@bjmu.edu.cn (H.C.); 2 Nutritional Epidemiology Group, School of Food Science & Nutrition, University of Leeds, Leeds LS2 9JT, UK; fshz@leeds.ac.uk; 3 School of Biological Sciences, Dublin Institute of Technology, D08 X622 Dublin, Ireland; 17888808890@163.com (P.A.); john.kearney@TUDublin.ie (J.K.)

**Keywords:** dietary perceptions, dietary acculturation, dietary change, international student

## Abstract

International students may have difficulties in dietary acculturation. This study aimed to evaluate the knowledge, attitude and practices (KAP) of diet and health during the acculturation of international students. A cross-sectional survey was conducted among a convenience sample of 473 international students in Dublin. Knowledge, attitude and practices towards diet and health were evaluated by a questionnaire with open- and closed-ended questions. It was found that 45.3% of participants had a broad concept of a healthy diet, while few knew its specific contents. Furthermore, 75.3% of participants could explain the term functional food, and among them, 62.1% knew the appropriate definition of functional food. Participants who perceived their health very good and excellent were more likely to believe that their health status was determined by their own control. The consumption rate of functional food varied among regions and South and Central America students had the highest usage rate (44.5%) and Asian students had the highest daily usage rate (52.7%). Participants who were younger, single, from African and South and Central American countries, or who were in Ireland for less than one year were more likely to report dietary change after immigration. In conclusion, insufficient knowledge and self-perception towards diet and health as well as unhealthily dietary changes exist among international students living in Dublin.

## 1. Introduction

International students or foreign students are those who travel to another country for an educational reason [1]. According to the United Nations Educational, Scientific and Cultural Organization (UNESCO), over 2.5 million students were enrolled outside their country of citizenship in 2009 and the number of international students might rise to approximately 7 million by the year 2020 [2]. In recent years, except the main destination countries like the United States, the United Kingdom and Germany, Ireland is gradually becoming a country with rapid growth in the number of international students after New Zealand and the Netherlands [3]. Based on the results of the 2006 Census in Ireland (23 April 2006), non-nationals made up 10% of the population in Ireland, and this figure was set to increase further in the coming years [4].

Despite several advantages of studying abroad (i.e., advanced knowledge and multicultural horizons) [5], dietary changes and health care problems are challenging and disturbing to international students. Dietary behavior played a major role in acculturation for international students [6]. A qualitative study showed that most international students were unsatisfied with the local foods they consumed [7]. Edwards et al. [8] found that many international postgraduate students experienced food neophobia (i.e., the phenomenon of rejection to eat unfamiliar or unknown food) [9] and the food neophobia scores were even increased after a three-month follow-up. The process of acculturation resulted in changes in health behavior, such as diet and drinking behavior, among international students [7]. As a result, body weight might increase during dietary acculturation, which could potentially impact their health if the weight gain continued [10]. Thus, it is important to pay attention to the dietary behavior and health of international students.

The concept of a healthy diet has been studied extensively and several methods of dietary assessment and healthy dietary patterns have been well developed, such as Mediterranean diet (MeDi), Dietary Approaches to Stop Hypertension (DASH), Healthy Diet Indicator (HDI), Healthy Eating Index (HEI) and so on [11]. Furthermore, adolescent dietary guidelines also varied slightly among different regions. For instance, the World Health Organization (WHO) encouraged adolescents and young adults to consume five main food groups (i.e., grains, fruits and vegetables, milk and dairy food, meats, and fishes) [12]. The American dietary guideline also recommended adolescents to consume limited solid fats, cholesterol, added sugars and refined grains [13]. The Australian dietary guideline stated that adolescents required a wide variety of nutritious foods [14]. Therefore, adolescent students or young adults from various regions may share different dietary knowledge due to individuality and a localized health environment. Previous studies showed that individual dietary knowledge affected one’s food choices that are also influenced by their health attitudes [15]. Thus, in order to understand international students’ dietary acculturation, their dietary knowledge and attitudes should be paid more attention.

The functional foods are a food group that has specific functions related to improving health or preventing disease beyond adequate nutritional effects, such as low-fat milk [16,17]. Consumer’s awareness and attitude towards functional foods differed among European countries and America [18]. Labrecque et al. reported that French awareness of functional foods is lower than that of Americans and Canadians [19]. Besides, compared to Americans who easily accepted and consumed functional foods [20], Europeans’ attitudes were more critical and doubtful [21]. However, awareness and acceptance among international students are still unclear, and whether they take consumption of functional foods as a way to cope with dietary acculturation is worthy of exploration.

A Knowledge, Attitude and Practices (KAP) survey is an effective method that records the declarative opinions or statements of participants using predefined questions formatted in standardized questionnaires to provide access to quantitative and qualitative information [22], and it is also an effective method of investigation in nutrition and diet research. A KAP survey found that diabetic patients with higher scores for knowledge of dietary control had better practices of controlling blood glucose with positive attitudes [23]. Although KAP surveys on diet, lifestyle or nutrition among medical students or other subgroups have been conducted [24,25], similar studies among international students have not been reported yet. To some extent, knowledge, beliefs and attitudes could affect health-related behaviors [26]. Students who studied abroad might experience dietary changes and have different food choices with various dietary knowledge and attitudes during acculturation. Furthermore, KAP investigation related to diet and health among them will provide clear information. However, no research data on dietary attitudes and habits of immigrants in Ireland are available. Thus, this study was conducted to evaluate the dietary knowledge and attitudes among international college students and explore how their diets changed after migration to Ireland.

## 2. Materials and Methods

### 2.1. Participants and Sampling

This cross-sectional study was conducted among international students from the upper-level English language classes. Inclusion criteria were students who were 18 years or above who were living in Ireland for at least one week and who were registered in the English language school (upper-level) at the time of the study. Students who did not have high English level or who could not understand the questionnaire well were excluded.

The Advisory Council for English Language Schools (ACELS) website was accessed and the database of recognized schools in Dublin was used as the sampling frame. Schools belonging to the ACELS were qualified and approved by the Education sector to deliver English language education. Students who attended these schools were mainly international students (i.e., students who moved from their mother country to Ireland) whose first language was not English. From a list of 60 recognized schools, 10 schools were randomly selected. Managers of these schools were invited to take part in this study via telephone calls. All schools contacted agreed to participate. Then the teachers of upper-level classes invited all the students who met the inclusion and exclusion criteria to participate in this study.

### 2.2. Ethical Statement

This study was approved by the Research Ethics Committee of Dublin Institute of Technology. Permission to conduct the study was also sought from ten selected language schools. Confidentiality and anonymity were explained to each participant, and oral consent was obtained from all participants.

### 2.3. Data Collection

The questionnaires and an instruction letter for the teachers of each class were sent to the participating schools. The researchers QZ and PA went to each school informed and explained the detailed procedure of the study to the teachers before the study. At the end of the class, the teachers distributed questionnaires to the students who agreed to participate and made a clear explanation of the purpose and requirement of this study. These participants completed the questionnaire and returned it to the teachers. Then the filled questionnaires were gathered and delivered to researchers by the managers of each school.

### 2.4. Questionnaires

The questionnaire used in this study was self-reported with closed-ended and open-ended questions and could be broadly divided into two parts. One was socio-demographics information, including age, gender, marital status, education level, nationality and length of current stay in Ireland. Nationalities were further grouped into four categories by geographic region, including Asia, Europe, Africa, and South and Central America. The other was healthy diet and functional food knowledge, self-perceived health status, the degree of “Health Locus of Control” (HLC) (i.e., an area-specific measure of locus of control expectancies developed to predict health-related behavior) [27], dietary change practices and consumption of functional food.

In the functional food section, participants were asked to choose the best explanation of the functional food from four choices (i.e., healthy food; artificial food; food with a specific benefit above the nutrients it naturally contains; and genetically modified food). The statement “food with a specific benefit above the nutrients it naturally contains” is the most accurate. For questions about attitudes and behaviors towards the functional food, the meaning and examples of functional foods were explained, which ensured that the participants understood the questions correctly.

Participants were asked to report their subjective health status and to express their opinions to three health-related attitudinal statements (HLC), based on a 4-point Likert scale (1 = strongly disagree; 2 = tend to disagree; 3 = tend to agree; 4 = strongly agree). The statement was tailored to meet our study aims, including “good health is mainly determined by chance, and there is not much that I can do to influence my long term health” (HLC-1), “my health is mainly controlled by outside influences over which I have little or no control” (HLC-2), and “my health is under my own control, and I can improve my long term health by adopting a healthy lifestyle” (HLC-3) [27]. Responses to HLCs were further divided into “agree” or “disagree” with the statements.

Questions concerning knowledge of healthy diet, main dietary changes (i.e., Compared to the diet you had in your home country, what are the major differences of your current diet in Ireland?) were open-ended. Furthermore, responses to these questions were grouped into approximately ten broad categories. Besides, functional food consumers were classified as those who reported consuming any food or drinks with added ingredients that claimed to improve their health.

The questionnaire took approximately 15 min to complete. It was reviewed for content validity and reliability by a panel of nutritionists and food scientists. The final questionnaire was pilot tested on 20 international undergraduate students studying in the Dublin Institute of Technology, to assess clarity, literacy and redundancy.

### 2.5. Statistical Analysis

Data analyses were performed using the SPSS v.15 software (IBM Corp. Armonk, NY, USA). Results were mainly presented as frequencies and percentages. Cross-tabulations and Pearson Chi-square analyses were conducted to explore differences of participants’ perception of health status, dietary changes and HLCs across demographic characteristics. If the cross-tabulation was a 2 × 2, Yates continuity correction was performed when interpreting *p* values. Furthermore, the level of 5% significance was used throughout the statistical analyses. Content analyses were performed by QZ and PA for open-ended questions. The researchers read all the answers and grouped them into categories individually. They then reviewed the groupings together and discussed to reach an agreement when inconsistency in the groupings existed.

## 3. Results

A total of 604 international students were invited to participate in the study, and 507 students responded and returned the questionnaire. The response rate of this study was 84%. Twenty participants with a serious lack of demographic information and fourteen participants who did not answer or irrelevantly answered most of the questions were excluded. Finally, information for 473 participants was obtained and these data were included in the analyses.

### 3.1. Sample Characteristics

Socio-demographic information was presented in Table 1. Among 473 participants, 43% were male and 57% were female. They have been in Ireland for one week up to 7.4 years (mean 66.8 ± 75.4 weeks). The proportions of participants in Ireland for less than one year (53.9%) and at least one year (46.1%) were nearly equivalent. The majority of the participants were between 18 and 26 years old (60.9%), were single (82.8%) and had tertiary education level (80.2%). Participants came from 36 countries, with the highest proportion of Asians (31.3%). Among the participants, 15.1% were suffering from chronic diseases such as brittle bones/osteoporosis, diabetes and high blood pressure.

### 3.2. Healthy Diet Knowledge

**Figure 1:**
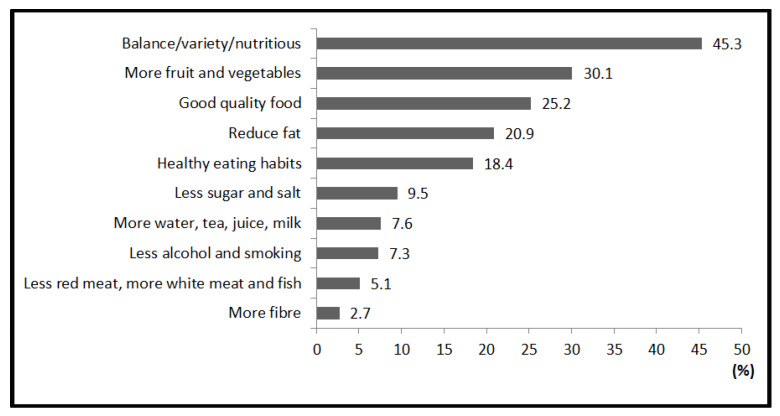
Ten categories of “healthy diet” defined by participants (*n* = 369). Reduce fat: includes less saturated fat, less fatty food, fast food or junk food. Good quality food: includes fresh, natural, organic food. More fiber: includes more cereal, brown bread; less white bread. Healthy eating habits: includes regular and proper meal time, proper amount of food.

The participants were asked to choose important factors influencing health and describe their perception of a healthy diet. Among the participants, 32.7% thought diet/food were important factors influencing health. When asked the meaning of a healthy diet, about 78% of participants provided appropriate descriptions or examples of a healthy diet; and 104 participants did not answer or answered incorrectly (22%). The statements about a healthy diet were grouped into 10 categories as presented in Figure 1. The majority regarded a healthy diet as “balance/variety/nutritious” (45.3%), followed by “more fruit and vegetables” (30.1%) and “good quality food” (25.2%). Furthermore, 20.9% of participants linked a healthy diet to “reduce fat”, and 18.4% noticed the importance of “healthy eating habits” to a healthy diet. “Less sugar and salt”, “more water, tea, juice and milk”, “less alcohol and smoking”, “less red meat, more white meat and fish” as well as “more fiber” were also mentioned (less than 10% in each category).

### 3.3. Health Status and Health Locus of Control Attitudes

As shown in Table 2, the participants (*n* = 465) generally perceived their health as good to excellent. Almost half of them reported good health status (45.8%). Differences in self-perceived health status were found among participants from different regions. Asian (61.5%) and African (46.0%) students were more likely to choose “good” to describe their health status, while European students were more likely to choose “very good” (49.6%). Besides, the proportion of students from South and Central America (16.6%) who perceived excellent health status was three times more than students from Asia (4.9%) (*p* < 0.001, Pearson Chi-Square analysis).

Attitude towards HLC varied among participants from different regions (Table 3). Asian and African students were more likely to agree with HLC-1 (39.8% and 26.2%, respectively) and HLC-2 (33.3% and 40.5%, respectively) than students from Europe or South and Central America (both less than 17%). Participants from Asia (19.0%) disagreed more with HLC-3 than those from other regions (less than 7%).

### 3.4. Perception and Consuming Practices of Functional Food

**Figure 2:**
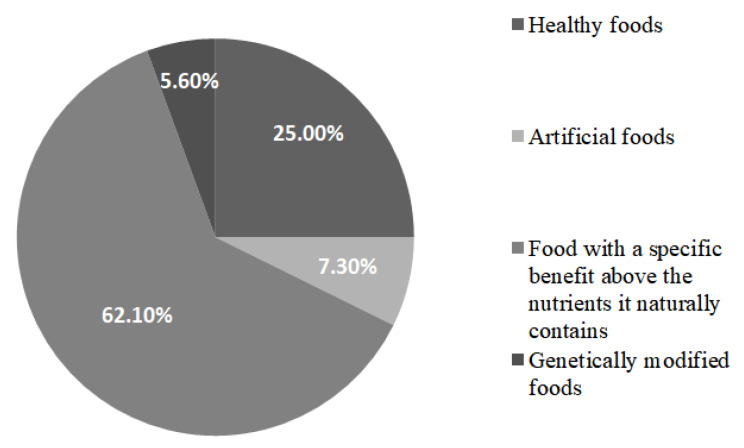
Participants’ interpretation of functional food (*n* = 356).

Among all participants, 75.3% can explain the term functional food (*n* = 356), but only 36.2% were aware of functional food and reported having heard this term before (*n* = 170). As shown in Figure 2, more than half of the participants (62.1%) selected “food with a specific benefit above the nutrients it naturally contains” as the best explanation of the term “functional food”. Furthermore, 25% interpreted functional food as “healthy food”. Only a small part of respondents thought of the “functional food” as “artificial food” (7.3%) or “genetically modified food” (5.6%).

As shown in Table 4, functional food consumption was associated to geographical region. The proportions of participants who consumed functional food from Asia and South and Central America (41.7% and 44.5%, respectively) were higher than those from Europe and Africa (25.7% and 21.3%, respectively) (*p* < 0.01, Pearson Chi-Square analysis). On the contrary, the participants from Europe and South and Central America were more specific in reporting functional foods consumption (data not shown).

As shown in Table 5, Asians accounted for the highest proportion (52.7%) in functional food daily consumption. Besides, the most popular functional food consumed was probiotic yogurts, followed by fortified juices with vitamins and fortified milk with vitamins (data not shown).

### 3.5. Dietary Changes after Moving to Ireland

All participants were required to answer whether or not their overall diet had changed since they came to Ireland. Table 6 showed that the majority of them (66.8%) perceived some dietary changes, compared with 26.3% reporting “no change”. Only 6.9% of the respondents had no idea of the change in their diet.

Significant differences were found when grouping by socio-demographic characteristics (i.e., age, marital status, regions, the length of living in Ireland) (Table 6). Participants in the younger age group were more likely to mention dietary changes. Compared with participants who were married or had been married, single participants were more likely to experience dietary change. More than 84% of South and Central Americans and Africans perceived dietary changes and less than 12% reported the opposite opinions. By contrast, Asians (39.0%) and Europeans (30.9%) were less likely to experience dietary change in Ireland. Furthermore, 10.6% of Asian participants did not know whether dietary change happened to them or not, which was more than twice as high as the percentage of Africa and South and Central America. Interestingly, people who lived in Ireland longer (i.e., one year and above) were less likely to experience dietary change.

**Figure 3:**
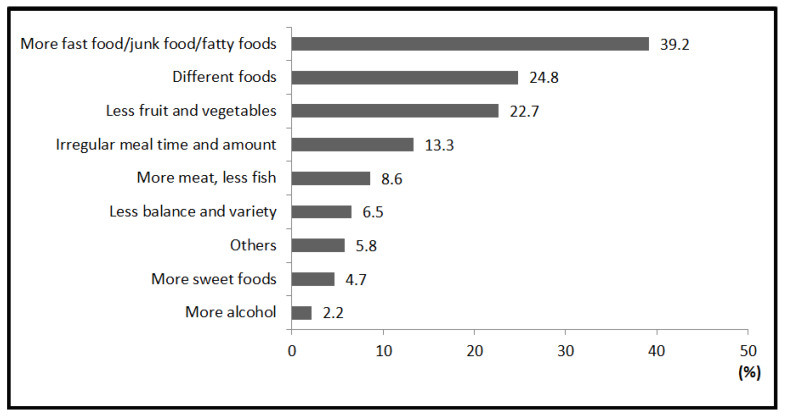
Nine groups of main dietary changes among international students in Ireland (*n* = 278). More fast food/junk food/fatty food: includes not fresh food, unhealthy food, bad quality food, artificial food and processed food. Different food: includes more bread/sandwich/potato, less staple food (e.g., rice, bean, and pasta) in home country. Irregular meal time and amount: includes change in time, amount and type of food in breakfast, lunch and dinner. Others: includes less meat, more fruit and vegetables, less alcohol, less fat, less amount of food eaten.

Among dietary changes, “more fast food/junk food/fatty food” was the most frequently stated change (39.2%), followed by “different food” (24.8%). Some participants declared that the consumption of certain types of their ethnic food (e.g., beans, rice) was reduced. “Less fruit and vegetables” ranked third as lots of participants complained that fruit and vegetables were very expensive in Ireland. There were 13.3% of participants who mentioned “irregular meal time and amount” as the main dietary change. According to them, this was due to part-time jobs, living with a host family, etc. The percentage of participants who referred to “more meat, less fish” (8.6%) was a little bit higher than that of those who mentioned “less balance and variety” (6.5%) and “more sweet food” (4.7%). Only 2.2% of participants reported “more alcohol” as the main change of diet. The group “others” accounted for only 5.8% of the total responses, including “less meat”, “more fruit and vegetables”, “less alcohol”, “less fat” or “less amount of food eaten” (Figure 3).

## 4. Discussion

The current study explored international students’ dietary knowledge, attitude and practices after immigrating to Ireland. This study included 473 participants with a relatively balanced gender ratio. Our major findings were as follows: (1) less than half of the participants had a broad concept of healthy diet and even fewer knew the specific meaning; (2) more than two thirds of the participants could explain the term functional foods, and Asian students had the highest usage and daily usage rate of functional foods; (3) participants who perceived their health better were more likely to think that their health status was controlled by themselves; (4) participants who were younger, single, from African and South and Central American countries or who were in Ireland for less than one year were more likely to report dietary change after immigration.

In our study, almost half of the international students had a broad concept about healthy diet (i.e., “balanced/various/nutritious diet”). However, the number of participants who related a healthy diet to “reduce fat” or “less sugar and salt” was less than those who related a healthy diet to “more fruit and vegetables". Participants who knew that a healthy diet is associated with “less red meat, more white meat and fish” and “more fiber” were fewer. Dietary knowledge survey results in this study indicated that dietary knowledge was not comprehensive among international students in Ireland. Previous research concluded that the awareness of a healthy diet was generally insufficient among international students, especially those studying nonhealth sciences [28]. Therefore, it is necessary to provide international students with essential knowledge of a healthy diet. The knowledge could be spread via English language schools by integrating dietary knowledge with other core subjects, such as language [29].

Functional foods that claim to improve health and wellbeing generally have special functions such as protecting the cardiovascular system, preventing cancers and so on [30,31,32]. Functional food is gradually being accepted by consumers, especially elderly adults [33]. However, whether international students take functional food as a way to keep healthy is worth exploring. Besides, the perception, acceptability or consumption of functional food among international students remains unclear. In the current study, functional food was found to be used by some international students, with differences among students from different regions. Our results showed that although the percentage was not high among participants who knew functional food, their perception was largely correct. The previous study suggested that increased knowledge was an important factor promoting functional food consumption [33]. Thus, it is suggested that the information of functional food could be incorporated into nutrition education for international students.

For the attitude of international students towards their health status and health locus of control (HLC), our results showed that participants who evaluated their health better were more likely to believe that their health status was determined by their own control (e.g., the European participants). By contrast, Asian participants who were least likely to deem their health status as very good or excellent showed no strong tendency to either agree or disagree with all attitudinal statements. Marques et al. also found that 20% of the total variance in people’s subjective health status was due to country differences [34]. Intriguingly, the consumption percentage and daily use of functional food among Asian students were high. Thus, it can be seen that whether international students adopted functional food or not was not determined by their own attitude towards health status. Furthermore, Herath et al. also came to similar conclusions [35]. The differences in subjective health status, attitudes towards health control and functional food consumption among different countries might be attributed to the distinctness of personality traits across countries. The Five-Factor Model (FFM) is used to describe human personality psychologically, which consists of five trait factors: Neuroticism (N); Extraversion (E); Openness (O); Agreeableness (A); and Conscientiousness (C) [36]. It showed that people from Asian countries were lower in Extraversion and higher in Neuroticism compared to people from European countries [37]. Besides personality, cultural diversity or climate differences might also be potential reasons; related research should be pursued. 

We found that international students may change their dietary practices to adapt to a new life abroad. In the current study, participants who were younger, single, from African and South and Central American countries and who were living in Ireland for less than one year were more likely to perceive overall dietary changes. However, the dietary changes they reported showed that their diet became unhealthier after coming to Ireland. As they reported, “more fast food/junk food/fatty food” was the most common change, and lower intakes of fruit, vegetables, and fish were also frequently reported. This result was consistent with the previous study showing that people from non-Western countries suffered from a worse dietary change after migrating to Western countries [38,39]. Furthermore, these dietary changes may be related to the increasing prevalence of diet-related diseases among the immigrants in certain ethnic groups, e.g., South-Asians in the UK [40]. Besides, Akresh et al. [41] found that Hispanic immigrants had a greater degree of dietary change with a longer stay in the United States, which is contrary to our results in which being in Ireland for less than one year was more likely to lead to dietary change. It is possible that newcomers were more likely to be influenced by the local Irish dietary culture and environment because of living with a host family, eating outside, etc. The longer they settled down, the more familiar they were with their ethnic food market in Ireland so that they might return to their original diet.

To the authors’ knowledge, literature on the diet of international students was limited. This study is novel in its examination of diet and health among international students who were living in Ireland for study. The current study included not only international students’ dietary knowledge and attitude but also their practices and dietary changes after immigrating. Besides, the participant characteristics of youth, bachelordom and high education level were in accord with the fast facts towards college and university students from the National Center for Education Statistics [42]. Therefore, the participating international students who came from 36 countries were representative and cross-cultural.

However, this study was limited by its cross-sectional nature and the convenience sample of international students from different countries. Our findings might not apply to students from other countries and immigrants with little English skills or who were working in Ireland. Moreover, no comparative studies on domestic students were conducted. We were unable to differentiate which changes were caused by migration to Ireland and which changes were caused by leaving one’s hometown or parental home for other cities. Future studies among Irish students who left their parental home or hometown for other cities for tertiary education are worth conducting. Besides, there is some variability in the samples as the length of staying in Ireland ranged from one week to 7.4 years. Information on participants’ socioeconomic and health status and religious information that might impact results were not assessed in the survey.

## 5. Conclusions

In conclusion, although international students had rough ideas about healthy diet and functional food, the specific knowledge they knew was insufficient. Self-perceived health status and attitude towards influencing factors of health differed significantly among geographical regions. Migration did influence the diet of international students and generally had unhealthy effects. Therefore, international schools and Irish society should be aware of the dietary and health status of international students during the process of acculturation. More dietary knowledge should be taught to international students to help them avoid adverse health effects.

## Figures and Tables

**Table 1:** Socio-demographic and health characteristics of the study participants.

Participant Characteristic	Respondents	
	*n*	%
Gender		
Male	203	43.0
Female	269	57.0
Age (years)		
18–26	387	60.9
>26	184	39.1
Education		
Primary/Secondary	93	19.8
Tertiary	377	80.2
Marital status		
Never married	389	82.8
Married/had been married	81	17.2
Region *		
Asia	144	31.3
Europe	141	30.7
Africa	52	11.3
South and Central America	123	26.7
Duration in Ireland		
<1 year	255	53.9
≥1 year	218	46.1
Health status		
Suffering from chronic diseases	60	15.1
Health	337	84.9

* Asia includes: China (including Hong Kong), India, Japan, Korea, Malaysia, Mongolia, Syria and Vietnam. Europe includes: Belarus, Croatia, Estonia, France, Germany, Hungary, Italy, Latvia, Novaya Zemlya, Poland, Russia, Serbia, Slovak Republic, Spain, Switzerland and Ukraine. Africa includes: Egypt, Madagascar, Mauritius and South Africa. South and Central America includes: Argentina, Bolivia, Brazil, Chile, Colombia, Cuba, Jamaica and Mexico.

**Table 2:** Self-perceived health status classified by geographical region.

	*n*	Poor	Fair	Good	Very Good	Excellent	*p* *
		*n* (%)					
Total participants	465	6 (1.3)	42 (9.0)	213 (45.8)	154 (33.1)	50 (10.8)	
Geographical region							
Asia	143	2 (1.4)	18 (12.6)	88 (61.5)	28 (19.6)	7 (4.9)	<0.001
Europe	139	0.0 (0)	6 (4.4)	48 (34.5)	69 (49.6)	16 (11.5)	
Africa	50	2 (4.0)	8 (16.0)	23 (46.0)	13 (26.0)	4 (8.0)	
South and Central America	120	2 (1.7)	9 (7.5)	47 (39.2)	42 (35.0)	20 (16.6)	

* Pearson Chi-Square analysis, relationship was deemed to be statistically significant when *p* < 0.05.

**Table 3:** Attitude towards Health Locus of Control (HLC) classified by geographical region.

	HLC-1 ^a^				HLC-2 ^b^				HLC-3 ^c^			
	*n*	Dis-agree (%)	Agree (%)	*p* *	*n*	Dis-agree (%)	Agree (%)	*p* *	*n*	Dis-agree (%)	Agree (%)	*p* *
Total	426	78.4	21.6		423	76.6	23.4		437	9.2	90.8	
Geographical region												
Asia	123	60.2	39.8	<0.001	120	66.7	33.3	<0.001	126	19.0	81.0	<0.001
Europe	132	91.7	8.3		134	85.1	14.9		136	3.7	96.3	
Africa	42	73.8	26.2		42	59.5	40.5		48	2.1	97.9	
South and Central America	120	85.8	14.2		118	83.9	16.1		117	6.8	93.2	

^a^ HLC-1, “good health is mainly determined by chance, and there is not much that I can do to influence my long term health”. ^b^ HLC-2, “my health is mainly controlled my outside influences over which I have little or no control”. ^c^ HLC-3, “my health is under my own control, and I can improve my long-term health by adopting a healthy lifestyle”. * Pearson Chi-Square analysis, relationship was deemed to be statistically significant when *p* < 0.05. (Disagree included strongly and tend to disagree; agree included strongly and tend to agree; don’t know was excluded).

**Table 4:** Functional food consumption classified by geographical region.

	*n*	Nonconsumer (%)	Consumer (%)	*p* *
Total participants	445	64.7	35.3	
Region				
Asia	139	58.3	41.7	0.001
Europe	140	74.3	25.7	
Africa	47	78.7	21.3	
South and Central America	119	55.5	44.5	

* Significance of relationships calculated by Pearson Chi-square analysis. Relationships were deemed to be statistically significant when *p* < 0.05.

**Table 5:** Functional food consumption frequency classified by geographical region.

	*n*	Frequency of Use		*p* *
		Daily (%)	1–5 Times per Week (%)	
Total participants	155	46.5	53.5	
Region				
Asia	55	52.7	47.3	0.593
Europe	37	37.8	62.2	
Africa	9	44.4	55.6	
South and Central America	54	46.3	53.7	

* Significance of relationships calculated by exact probability analysis. Relationships were deemed to be statistically significant when *p* < 0.05.

**Table 6:** Perceived dietary change of international students in Ireland.

	*n*	No Change	Yes, it Has Changed	Don’t Know	*p* *
		%			
Total participants	464	26.3	66.8	6.9	
Age (years)					
18–26	282	20.6	73.0	6.4	0.001
>26	180	35.6	56.7	7.8	
Marital status					
Never married	380	23.7	69.7	6.6	0.020
Married/had been married	81	38.3	54.3	7.4	
Region					
Asia	141	39.0	50.4	10.6	<0.001
Europe	139	30.9	61.9	7.2	
Africa	51	11.8	84.3	3.9	
South and Central America	120	10.8	85.8	3.3	
Duration in Ireland					
<1 year	253	20.2	73.5	6.3	0.003
≥1 year	211	33.6	58.8	7.6	

* Significance of relationships calculated by Pearson Chi-square analysis. Relationships were deemed to be statistically significant when *p* < 0.05.
